# Prevalence, Potential Predictors, and Genotype-Specific Prevalence of Human Papillomavirus Infection among Sexually Active Students in Japan

**DOI:** 10.1371/journal.pone.0132462

**Published:** 2015-07-15

**Authors:** Hirohisa Imai, Hiroyuki Nakao, Hisae Shinohara, Mutsuko Watarai, Noriko Matsumoto, Takuya Yamagishi, Masuko Saito, Tadaichi Kitamura

**Affiliations:** 1 Department of Epidemiology and Health Policy, National Institute of Public Health, Wako-shi, Saitama, Japan; 2 Department of Epidemiology, Miyazaki Prefectural Nursing University, Manabino, Miyazaki, Japan; 3 Faculty of Education and Culture, University of Miyazaki, Gakuenkibanadainishi, Miyazaki, Japan; 4 Division of Nursing, Faculty of Healthcare, Tokyo Healthcare University, Shinagawa-ku, Tokyo, Japan; 5 Infectious Disease Surveillance Center, National Institute of Infectious Disease, Sinjuku-ku, Tokyo, Japan; 6 Department of Nursing, Teikyo University of Science, Adachi-ku, Tokyo, Japan; 7 Department of Urology, Asoka Hospital, Koto-ku, Tokyo, Japan; Istituto Nazionale Tumori, ITALY

## Abstract

**Background and Methods:**

We conducted a community-based study to evaluate genotype-specific prevalence of high-risk HPV (HR-HPV) and potential predictors of its presence in young, asymptomatic, female college students. Self-administered surveys and vaginal swabs for self collection were distributed to students of participating schools. A sufficient cellular component in cervical samples was verified by examining for the presence of human β-globin DNA by PCR. A total of 1,118 valid cervical samples were subjected to screening for HR-HPV infection with the Digene Hybrid Capture 2 assay, followed by identification of HPV genotypes with GENOSEARCH HPV31 kit. Logistic regression was used to adjust for confounding factors associated with HR-HPV positivity and the adjusted odds ratio (AOR) was calculated.

**Results:**

The median age of recruited students was 20 years. Of the 1,118 women who provided valid cervical samples for testing, 770 had sexual intercourse in the past, of which 125 (16.2%) were positive for HR-HPV. Logistic regression analysis revealed that HR-HPV infection was associated with smoking history (AOR 2.13; 95% confidence interval [CI] 1.98 to 5.05; *p* < 0.01), total number of partners (AOR 4.72; 95% CI 1.97 to 11.32 if > 5 partners; *p* < 0.001), number of partners in the past 6 months (AOR 3.12; 95% CI 1.42 to 6.87; *p* < 0.01), improper use of condoms (AOR 2.21; 95% CI 1.25 to 3.90; *p* < 0.01), and chlamydia infection (AOR 2.61; 95% CI 1.28 to 5.34; *p* < 0.01). The most common HR-HPV genotype was type 52 (6.4%), followed by 16 (3.1%), 56 (3.0%), and 58 (2.6%).

**Conclusion:**

Compared with previous reports in East Asian coutries, the prevalence of HR-HPV infection among young, asymptomatic, female students before the nationwide use of vaccination in Japan was in the intermediate range. The most common HR-HPV genotypes were HPV 52, 16, 56, and 58.

## Background

Cervical cancer ranks as one of the most common cancers affecting women in most countries. A total of 250,000 women die of cervical cancer each year worldwide [1]. In Japan, approximately 10,000 women are diagnosed with cervical cancer every year, and approximately 2,500 of them decease each year [2]. Human papilloma virus (HPV) infection predisposes women to cervical cancer. Only high-risk genotypes (HR-HPV) cause cervical cancer, while low-risk genotypes are rarely associated with cervical cancer or other neoplasms [3].

Data accumulated by long-term follow-up studies indicate that infection of 12 HPV types (16, 18, 31, 33, 35, 39, 45, 51, 52, 56, 58, 59) classified as class I (established) carcinogens were more common in women with cervical cancers than those without cervical lesion. Several other rare HPV types (eg, HPV26, 67, 68, 69, 73, 82) were also found more commonly in women with cervical cancers [4]. Approximately 20% of women with persistent cervical HR-HPV infection for 1 year will develop cervical intraepithelial neoplasia or invasive cancer within 5 years [5]. New infections clear within 2 years in nearly all infected patients [6], but in a portion of infected patients, the persisting infection predisposes them to the development of cervical neoplasia. HPV vaccines were thus developed to prevent HPV infection, and vaccinations have been adopted worldwide ever since their approval by the FDA in 2006 [7]. Epidemiological information on general and genotype-specific prevalence of HR-HPV and potential predictors of infection are essential for further advances in the development of HPV vaccines and prevention of cervical cancer.

HPV infection is acquired through sexual intercourse and therefore, the sexual behavior of young people has a significant impact on HPV infection. Therefore, community-based, baseline epidemiological data, including HPV genotypes, in young women who are at a particularly high risk of HR-HPV infection are indispensable for monitoring the impact of vaccination. However, previous epidemiological studies were hospital-based and no community-based epidemiological information is available at present. The purpose of our study was therefore to elucidate the prevalence, potential predictors, and genotype-specific prevalence of HR-HPV among female students at colleges and vocational schools in a prefecture in southern Japan.

## Methods

### Study Design and Subjects

This was a community-based, cross-sectional study investigating asymptomatic HR-HPV infection in young women. There are 6 universities and 18 vocational schools in the city of Miyazaki, Japan. Initial inquiries were sent to the administrative offices of these schools to find out their willingness to participate in the study. Among those, 3 universities and 6 vocational schools agreed to participate in the study, and female students attending those schools who were 18 years or older were potential candidates. An investigator visited the participating schools to present the purpose and design of the study to students. Student health personnel of the schools also solicited participation and campus posters were used to promote the study. Written consent was obtained from each voluntary participant prior to distribution of study material. The study was conducted from October 2011 to February 2012 and was approved by Tokyo Healthcare University Institutional Review Board.

### Study Methods

The aim of the study was to assess the prevalence of asymptomatic HPV infection. Therefore, candidates who reported that they were aware of urogenital symptoms suggestive of or were being treated for sexually transmitted disease were excluded from the study. We distributed survey sets consisting of an envelope containing a self-administered blank questionnaire on sexual behavior, a HPV test kit, a chlamydia test kit, and seals stamped with an ID no. (set of four with the same number) to candidate students. The questionnaire on sexual behavior asked about the history of past sexually transmitted diseases, age at first sexual intercourse, total number of sexual partners, number of sexual partners in the past half year, and use of condoms. The other questions included in the questionnaire were smoking and drinking habit. Candidates were instructed to apply seals to the two test kits and the questionnaire, and to keep the one remaining seal as their personal ID no. To access the test results, participants were instructed to log in on the study website by cell phone or computer approximately 3 months after submitting the HPV and chlamydia test kits, and to retrieve the test results using their personal ID no. To completely to protect the privacy of the study participants, they were not contacted about treatment, but general information on HR-HPV infection and cervical cancer, and prevention, diagnosis, and treatment were posted on the website where the test results were provided.

### Specimens and Laboratory Methods

Study participants were instructed to obtain the cervical specimen for the HPV test by rubbing the cervix with a cotton swab included in the Cervical Sampler (Digene, Gaithersburg, MD). The cotton swab was then placed in a tube filled with Specimen Transport Medium. Participants were instructed to avoid handling the capped tube roughly or placing it in a hot environment prior to sample submission. Subjects were also told to collect the urine sample on the first urination in the morning for chlamydia testing. The cervical specimens and urine samples were to be taken to school by midmorning and placed in a refrigerated box and kept refrigerated at 2 to 8°C. The collected samples were carefully packaged and shipped refrigerated to a central laboratory in Tokyo. The specimens and samples were processed for testing within 24 hours after being submitted. HR-HPV infection in the samples was evaluated in two steps. First, the presence of HR-HPV was tested by the Hybrid Capture 2 (HC2) method (Digene). The denatured samples were mixed with a RNA probe mixture that contains probes for 13 HPV types known to be high-risk types, and the DNA/RNA complex was detected by immunoassay using an antibody detecting the DNA:RNA hybrids. Specific HPV genotypes were then identified with the samples that were positive for HR-HPV by HC2, using the GENOSEARCH HPV31 kit (Medical and Biological Laboratory, Nagoya, Japan). The kit can identify the following 31 HPV genotypes: 6, 11, 16, 18, 26, 31, 33, 35, 39, 42, 44, 45, 51, 52, 53, 54, 55, 56, 58, 59, 61, 62, 66, 68, 70, 71, 73, 82, 84, 90, and CP6108, utilizing reverse sequence-specific oligonucleotide PCR (PCR rSSO). A probe detecting a portion of genomic DNA that encodes human β-globin was also included as an internal control to verify presence of sufficient cellular component in the samples. Briefly, DNA extracted using QIAsymphony DSP viral/pathogen kit (Qiagen) from 400 μl of the sample that had been alkaline-denatured for the HC2 assay was amplified by multiplex PCR for 31 HPV DNA using biotin-labeled specific probes in 96-well Gene Amp PCR system 9700 (Life Technologies, Tokyo, Japan) with the following program: 35 cycles of denaturing at 94°C (30 sec), annealing at 61°C (1 min) and extension at 72°C (30 sec). The resulting amplicon mixtures were hybridized with fluorophore-loaded beads, followed by detection with phycoerythrin-bound streptavidin by the Luminex System (Luminex, Austin TX, USA).

Chlamydia DNA in urine samples was simultaneously assayed by SDA (Strand Displacement Amplification, Becton Dickinson).

### Statistical Analysis

Associations between HR-HPV positivity and its potential predictors were evaluated by univariate analysis using Pearson’s chi-square tests or Wilcoxon rank-sum tests. Logistic regression analysis was used to adjust for potential confounding factors associated with HR-HPV positivity. All covariates were entered into the model, and analyses were executed using stepwise variable selection. Odds ratios were estimated for each independent potential predictor and 95 percent confidence intervals were calculated after adjusting for all other variables retained in the model. A two-sided significance level of 0.05 was used. All statistical analyses were performed using Statistical Package for Social Sciences (SPSS, version 19).

## Results

The sample collection kits and questionnaire sets were distributed to 1,627 candidates. Among them, 1,183 subjects whose cervical sample contained human β-globulin DNA and who answered the questionnaire properly were analyzed. A total of 770 of these women had experience of sexual intercourse (Table 1) and they were defined as sexually active women. There were less young individuals, more smokers, and more drinkers in the sexually active group compared with the not sexually active group. All individuals who had no experience of sexual intercourse were negative for HPV and chlamydia. Intriguingly, 4 women who were reportedly not sexually active stated that they had a previous history of a sexually transmitted disease.

**Table 1:** General and sexual behavioral characteristics of respondents.

Characteristic	Women total* (n = 1,183)		Sexually active (n = 770)		Sexually inactive (n = 413)	
	n	%	n	%	n	%
**Age**						
18	104	8.8%	56	7.3%	48	11.6%
19	335	28.3%	167	21.7%	168	40.7%
20	311	26.3%	199	25.8%	112	27.1%
21–22	259	21.9%	182	23.6%	77	18.6%
23+	170	14.4%	163	21.2%	7	1.7%
**History of any STDs**						
Yes	58	4.9%	54	7.0%	4	1.0%
No	1,125	95.1%	716	93.0%	409	99.0%
**Smoking**						
Yes	111	9.4%	102	13.2%	9	2.2%
No	1,070	90.4%	666	86.5%	404	97.8%
**Drinking**						
Yes	729	61.6%	531	69.0%	198	47.9%
No	451	38.1%	236	30.6%	215	52.1%
**Age at first intercourse**						
≦15			106	13.8%		
16–17			302	39.2%		
18–19			268	34.8%		
20+			92	11.9%		
**Number of sexual partners in lifetime**						
1			225	29.2%		
2			147	19.1%		
3–4			171	22.2%		
5+			225	29.2%		
**Number of sexual partners in previous six months**						
No partner			149	19.4%		
1			475	61.7%		
2+			143	18.6%		
**Condom use**						
Always, throughout the entire sexual encounter			265	34.4%		
Always, but not from the beginning			55	7.1%		
Inconsistent condom use			446	57.9%		

Sexually active: having experienced sexual intercourse, Sexually inactive: never having experienced sexual intercourse

STD = sexually transmitted disease

**Fig 1:**
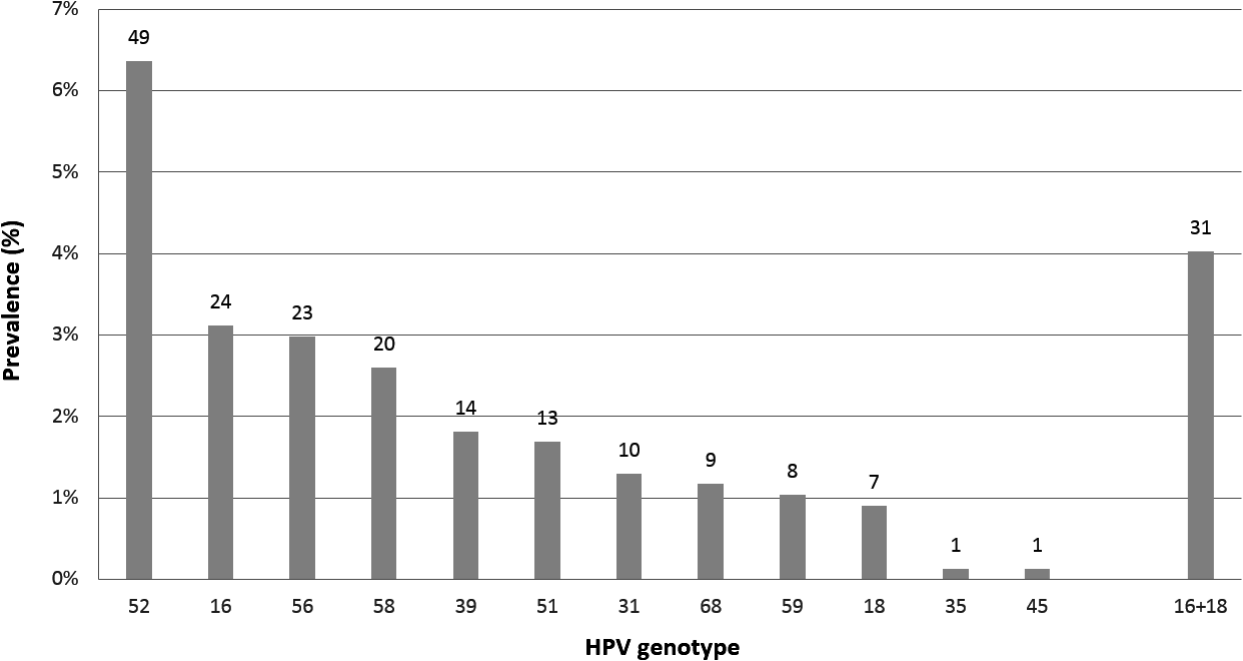
Prevalence of high-risk HPV by genotype among 770 sexually active subjects. Using the cervical specimens that participants had collected by themselves, HPV genotypes were analyzed using Hybrid Capture 2 assay (Digene/Qiagen) and GENOSEARCH HPV 31 (Medical & Biological Laboratories, Nagoya, Japan). Numbers on the x-axis indicate genotypes. The number on the top of the column indicates the number of women infected with high-risk HPV of the specific genotype.

A total of 125 women (16.2% of the sexually active subjects) had HR-HPV infection (Table 2). The most common HR-HPV genotype was HPV 52 (prevalence of 6.4%), followed by HPV 16 (3.1%) and HPV 56 (3.0%). The prevalence of HPV 16 and 18 was 3.1% and 0.9%, respectively, and no one was positive for both types (Fig 1). Twenty-four women were positive for 2 HR-HPVs, 12 women had 3 HR-HPVs, and 2 had 4 HR-HPVs. Chlamydia was positive in 44 women (5.7% of sexually active subjects).

**Table 2:** Prevalence of HR-HPV infection, and univariate and multivariate logistic regression analyses of the association between potential predictors and HR-HPV infection in sexually active respondents (n = 770).

Potential predictors	Univariate analysis		Multivariate analysis	
	HR-HPV-positive (%)	P value	AOR (95%CI)	P value
**Total**	**125 (16.2)**			
**Age**				
18	6 (10.7)	<0.05*	1.06 (0.33–3.36)	0.93
19	26 (15.6)		1.34 (0.64–2.78)	0.44
20	23 (11.6)		1.00 (0.49–2.05)	0.99
21–22	37 (20.3)		1.89 (1.00–3.60)	0.05
23+	33 (20.2)		reference	
**History of any STDs**				
Yes	13 (24.1)	0.11†	1.00 (0.47–2.10)	0.99
No	112 (15.6)		reference	
**Smoking**				
Yes	34 (33.3)	<0.001†	2.13 (1.20–3.75)	<0.01
No	91 (13.7)		reference	
**Drinking**				
Yes	91 (17.1)	0.34†	0.82 (0.51–1.33)	0.43
No	34 (14.4)		reference	
**Age at first intercourse**				
≦15	13 (12.3)	0.92*	0.30 (0.11–0.79)	<0.05
16–17	56 (18.5)		0.79 (0.37–1.71)	0.55
18–19	41 (15.3)		1.05 (0.50–2.22)	0.90
20+	14 (15.2)		reference	
**Number of sexual partners in lifetime**				
1	10 (4.4)	<0.001*	reference	
2	15 (10.2)		1.89 (0.79–4.52)	0.15
3–4	36 (21.1)		3.90 (1.74–8.71)	<0.001
5+	64 (28.4)		4.72 (1.97–11.32)	<0.001
**Number of sexual partners in previous six months**				
No partner	11 (7.4)	<0.001*	reference	
1	65 (13.7)		1.60 (0.79–3.24)	0.20
2+	49 (34.3)		3.12 (1.42–6.87)	<0.01
**Condom use**				
Always, throughout the entire sexual encounter	19 (7.2)	<0.001*	reference	
Always, but not from the beginning	6 (10.9)		1.24 (0.45–3.41)	0.68
Inconsistent condom use	99 (22.2)		2.21 (1.25–3.90)	<0.01
**CT-positive**				
Yes	20 (45.5)	<0.001†	2.61 (1.28–5.34)	<0.01
No	105 (14.5)		reference	

CT = Chlamydia trachomatis; STD = sexually transmitted disease; AOR = Adjusted odds ratio

*Wilcoxon rank-sum test (for trend)

†Chi-square test

The following factors were found to be associated with HR-HPV infection by univariate analysis: older age, smoking history, number of sexual partners (total and the number in the past 6 months), inappropriate use of condoms, and chlamydia positivity. Age at first intercourse was not significantly associated with HR-HPV infection.

Logistic regression analysis of the relationship of each factor with HR-HPV infection revealed that HR-HPV infection was associated with smoking history (adjusted odds ratio [AOR] 2.13; 95% confidence interval [CI] 1.98 to 5.05; *p* < 0.01), total number of partners (AOR 4.72; 95% CI 1.97 to 11.32 if > 5 partners; *p* < 0.001), number of partners in the past 6 months (AOR 3.12; 95% CI 1.42 to 6.87; *p* < 0.01), inappropriate use of condoms (AOR 2.21; 95% CI 1.25 to 3.90; *p* < 0.01), and chlamydia infection (AOR 2.61; 95% CI 1.28 to 5.34; *p* < 0.01) (Table 2).

## Discussion

Most studies identifying the prevalence of HR-HPV infection have thus far been conducted in visitors to medical institutions or health centers and may have been biased. Accurate epidemiological information on HPV infections, including genotype-specific prevalence, is essential for achieving further progress in prevention, early detection and appropriate treatment of cervical cancer, and therefore, population- or community-based studies are desired. In this study, the subjects were sexually active young women attending educational institutions in a relatively closed community. Women who had urogenital symptoms at the time of the questionnaire survey were excluded (no cytology was performed). The prevalence of HR-HPV infection (16.2%) in this study was close to the HR-HPV prevalence of 18.5% reported in a recent UK community-based study of young female students [8]. Compared with studies in other Asian countries, the overall prevalence in the present study lies between the prevalence of 23.2% in the 20–29 years age group in Korea [9] and the prevalence of 11.5% in the 18–24 years age group in a population-based study in China [10], except for the lower prevalence in the group of 18-year-olds in this study.

The AOR for smoking history was 2.13. While several previous studies reported that smoking history is a potential predictor [11–14], others did not find any association between smoking history and HR-HPV prevalence [12,15–17]. As reported elsewhere [8,11,13,15,17], the OR for HR-HPV positivity was higher if subjects had more partners (either numbers in lifetime or numbers in the past 6 months). The association in our study appeared stronger (AOR for subjects with 5 or more partners, 4.72) than that in other reports (OR for a similar number of partners was in the range of 1.1–3.9). The number of new sexual partners has also been reported as an independent potential predictor for the presence of HPV [15]. Because the subjects of our study were young women, it is reasonable to assume that the majority of relationships of these women with their sexual partners would have been relatively short, and that subjects with multiple partners would have been exposed to several new partners in a relatively short period, exposing them to even higher risk of HR-HPV infection. Previous reports suggest that condoms can only partially prevent the spread of HPV [18]. Supporting that notion, inappropriate and/or inconsistent condom use was found to weakly but significantly increase AOR for HR-HPV positivity. Chlamydia infection has been shown to correlate with HPV infection in several studies [8,12]. Because the present study was a cross-sectional study, the temporal relationship between chlamydia infection and HR-HPV infection was not evaluated, but there were more HR-HPV subjects in the chlamydia-positive group in the present study, suggesting that chlamydia infection is a potential risk factor for acquiring HPV. Age was not found to be a potential predictor in the present study. Several studies have reported no association between age and HPV prevalence [8,11], but others have reported that HPV prevalence is higher in younger women [13–15]. Several studies have also shown that age correlated with infection clearance, and they concluded that the older the patient, the more difficult it is to achieve clearance [6,9]. In the present study, however, no association between age and HR-HPV prevalence was found, which might be due to the limited age range that was examined.

HPV 52 genotype was the most common (6.4% of all sexually active subjects), followed by HPV 16 (3.1%), HPV 56 (3.0%), and HPV 58 (2.6%). The prevalence of HPV 18 was only 0.9%. The combined prevalence of HPV 16 and 18 in this study was 4.0%, but appears to vary between regions, being 1.5% and 0.8% in the US [19], 4.2% and 1.4% in the general population in the UK [15], 5.4% and 2.3% in a study of college students in the UK [8], and 6.4% and 0.6% in Estonia, respectively [20]. In our study, HPV 18 prevalence was particularly low. Further study is needed to determine whether this was due to regional factors or whether another reason was involved.

The present study has several limitations. First, the effective sample size of 1,183 subjects was not large. The number of potential candidates was limited by study design as we aimed to conduct a community-based study. Also, our approach was to recruit students in the area, excluding women who were not attending schools. This may have caused selection bias. Our findings should therefore be interpreted with caution. Second, the accuracy of self-reporting is limited. Four of the subjects who reported that they have never had sexual intercourse also reported a history of sexually transmitted disease, implying a possible error in interpreting the question or false entry in the questionnaire. It is impossible to fully eliminate possible bias due to misunderstanding of questions and/or false answers in the self-administered questionnaire, especially when questions are related to sexual behavior. For example, in a previous study that involved more than 10,000 subjects, 1.4% (13 of 914) of participants who reported having had no vaginal sex were chlamydia-positive [21]. Memory bias could also lead to inaccuracy of the survey [22]. Nonetheless, we believe that such bias minimally affected the findings in the present study, since none of the 4 subjects whose STD history was inconsistent with their sexual inactivity were positive either for HR-HPV or chlamydia.

## Conclusions

This study provided community-based, basic epidemiological information on HPV infection and genotype-specific prevalence in young women. These findings will hopefully be used to track changes in HPV prevalence and HPV genotype-specific prevalence, as well as changes in the prevalence of HPV-induced cervical cancer with the introduction of HPV vaccines in Japan.
